# The Roles of PI3K/AKT/mTOR and MAPK/ERK Signaling Pathways in Human Pheochromocytomas

**DOI:** 10.1155/2016/5286972

**Published:** 2016-11-20

**Authors:** Juan Du, Anli Tong, Fen Wang, Yunying Cui, Chunyan Li, Yushi Zhang, Zhaoli Yan

**Affiliations:** ^1^Department of Endocrinology, Key Laboratory of Endocrinology, Ministry of Health, Peking Union Medical College Hospital, Chinese Academy of Medical Sciences and Peking Union Medical College, Shuaifuyuan No. 1, Dongcheng District, Beijing 100730, China; ^2^Department of Urology, Peking Union Medical College Hospital, Chinese Academy of Medical Sciences and Peking Union Medical College, Beijing 100730, China; ^3^Department of Endocrinology, Affiliated Hospital of Inner Mongolia Medical University, Hohhot 010050, China

## Abstract

*Objectives*. The roles of PI3K/AKT/mTOR and MAPK/ERK pathways involved in the pathogenesis of pheochromocytoma and paraganglioma (PPGL) were demonstrated mostly by* in vitro* studies with rat or mouse cells and were mainly studied at transcriptional level. This study aimed to investigate the effect of these pathways on the proliferation of human PPGL cells and the activation of these pathways in PPGLs.* Methods*. Human PPGL cells were treated with sunitinib and inhibitors of PI3K (LY294002), MEK1/2 (U0126), and mTORC1/2 (AZD8055). Cell proliferation was detected by MTT assay. Protein phosphorylation was detected by Western blotting.* Results*. In most PPGLs, AKT, ERK1/2, and mTOR were activated. LY294002 (10 *μ*M), U0126 (10 *μ*M), AZD8055 (1 *μ*M), and sunitinib (1 *μ*M) inhibited PPGL cell proliferation in ten primary cultures of tissues, including four from patients with gene mutations. MEK1/2 inhibitor decreased mTOR phosphorylation. Inhibition of mTOR reduced phosphorylation of AKT and ERK1/2. Sunitinib inhibited phospho-ERK1/2 and phospho-mTOR.* Conclusion*. Our study suggested that PI3K/AKT/mTOR and MAPK/ERK signaling pathways play vital roles in human PPGL and are activated in most PPGLs. Inhibiting multiple pathways might be a novel therapeutic approach for PPGLs.

## 1. Introduction

Pheochromocytomas and paragangliomas (PPGLs) are neuroendocrine tumors arising from adrenomedullary chromaffin cells and extra-adrenal chromaffin cells of the sympathetic or parasympathetic ganglia. The tumors are associated with life-threatening complications due to their ability to release catecholamines—norepinephrine, epinephrine, and dopamine [1]. Once the diagnosis is established, the therapy of choice is radical resection.

Most PPGLs are benign and surgically curable, but when they are malignant, recurrent, or irremovable few effective therapies are available [2]. Intensive studies on PPGL tumorigenesis have led to the development of targeting drugs designed to improve the outcome of the tumors. PPGLs are generally divided into two major clusters [3–9]. Cluster 1 includes the tumors with von Hippel-Lindau (VHL) gene and the subunits of the succinate dehydrogenase (SDHx) mutation that lead to dysregulation of Krebs cycle and activation of hypoxia signaling pathway [10]. Cluster 2 involves the tumors with the mutation of rearranged during transfection (RET), neurofibromin 1 (NF1), kinesin family member 1B (KIF1Bb), transmembrane protein 127 (TMEM127), and MYC-associated factor X (MAX), which are associated with abnormal activation of phosphatidylinositol 3′ kinase (PI3K)/protein kinase B (AKT), mitogen-activated protein kinase (MAPK)/extracellular signal-regulated kinase (ERK), and mammalian target of rapamycin C1 (mTORC1)/p70S6K [11].

Recent studies on the prosurvival molecular pathways such as PI3K/AKT/mTOR and MAPK/ERK revealed that they play important roles in tumorigenesis of a wide array of tumors, including PPGLs [12]. The role of MAPK pathway in the pathogenesis of PPGL has been documented by a number of studies [13, 14]. Upregulated mRNA expression involving MAPK signaling pathway has been observed in the RET/NF1/TMEM127-related PPGL tissues [15]. In addition, elevated phosphorylation of AKT in sporadic PPGLs was found by using Western blotting and immunohistochemical detection [16]. Moreover, inhibition of PI3K/AKT pathway could effectively suppress proliferation of rat pheochromocytoma PC12 cells* in vitro *[17]. mTOR is a serine/threonine kinase involved in the regulation of cell growth and proliferation and consists of two separate protein kinase complexes, mTORC1 and mTORC2 [18]. The dysregulation of the mTOR signaling pathway was found to be associated with human tumors, including PPGLs [19]. Recently, Giubellino et al. found that the expression of mTOR (mTORC1 and mTORC2) mRNA was higher in PPGLs with SDHB and VHL mutations than in normal adrenal medulla [20]. AZD8055, a newly identified inhibitor targeting both mTORC1 and mTORC2, was found to be able to significantly inhibit both proliferation and migration of mouse PPGL cells* in vitro*, suggesting that targeted inhibition of mTOR promises to be a novel therapeutic alternative for PPGLs [20].

Mounting evidence has shown that the aforementioned pathways play significant roles in the pathogenesis of PPGLs, demonstrated mostly by* in vitro* studies with rat or mouse cells. Moreover, signaling pathways involving PPGLs were mainly observed at transcriptional level and little is known about the activation and roles of these pathways in human PPGLs. In addition, no studies have been conducted concerning the cross talk between these two vital cellular pathways, PI3K/AKT/mTOR and MAPK/ERK, in PPGLs. In this study, we examined the effect of PI3K/AKT/mTOR and MAPK/ERK pathways on the growth of human PPGL cells and their interactions. We also observed the phosphorylation level of key proteins in these pathways in PPGLs with different gene mutations.

## 2. Materials and Methods 

### 2.1. Reagents

LY294002 (PI3K inhibitor) and U0126 (MEK1/2 inhibitor) and 3-(4,5-dimethylthiazol-2-yl)-2,5-diphenyltetrazolium bromide (MTT) and bicinchoninic acid (BCA) kit were purchased from Sigma-Aldrich (St. Louis, MO, USA). AZD8055 (mTORC1/2 inhibitor) and sunitinib (a multitargeted receptor tyrosine kinase inhibitor) were procured from Selleck (Houston, Texas, USA). The PPGL cells were maintained in Dulbecco's modified Eagle's medium (DMEM) supplemented with 15% fetal bovine serum (FBS) and 50 units/mL penicillin/50 mg/mL streptomycin (Gibco-Life Technologies, Grand Island, NY, USA). Antibodies against phospho-AKT (Ser473), phospho-p44/42MAPK (ERK1/2) (Thr202/Tyr204), and phospho-mTOR (Ser2448) were obtained from Cell Signaling Technology (Beverly, MA, USA). *β*-Actin antibody was from Sigma-Aldrich (St. Louis, MO, USA). Secondary antibodies were horseradish peroxidase-conjugated goat anti-mouse IgG (MultiSciences, Beijing, China) used for p-AKT, p-ERK1/2, and *β*-actin and goat anti-rabbit IgG (MultiSciences, Beijing, China) for p-mTOR. Protease inhibitor cocktail was from Roche (Indianapolis, IN, USA). Tissue protein extraction reagent was purchased from Applygen Technologies (Beijing, China).

### 2.2. Human Tumor Samples

Tumor samples were collected during surgical excision. Fresh tumor tissues were immediately treated for cell culture or frozen for Western blot study. PPGLs were histopathologically identified. All patients were screened for germline mutations of VHL, RET, SDHB, SDHC, and SDHD genes. In addition, nine nonmetastatic PPGLs (3 with RET mutation, 3 with VHL mutation, and 3 with SDHB mutation) were harvested for Western blotting. Clinical data of the patients are presented in Table 1. Informed consent was obtained from all subjects and the study was approved by the PUMCH Ethics Committee for Human Research with an IRB approval number of S-K084.

### 2.3. Cell Culture

Human PPGL cells were primarily cultured as previously described [21]. In brief, human PPLG cells were taken by sequential collagenase (type I) digestion from PPGL tissues and incubated in DMEM supplemented with 15% FBS and antibiotics at 37°C in 5% CO_2_ atmosphere. Cells were allowed to adhere and grew fully under standard conditions.

### 2.4. Drug Treatment and Protein Extraction

Human PPGL cells were plated into 6-well plates at a density of 10^5^ cells/well and were allowed to grow until they were 80% confluent. Then, cells were treated to determine the optimal stimulation time and the optimal concentration of the inhibitors as follows: (1) after serum starvation for 6 hours, cells were treated with the serum (the final concentration of serum in medium was 15%) for different incubation periods (5, 15, 30, and 60 min). (2) Serum-starved cells were pretreated with increasing concentrations of selective antagonists, including LY294002 (0.1, 1, 5, and 10 *μ*M), U0126 (0.1, 1, 5, and 10 *μ*M), AZD8055 (0.01, 0.1, 1, and 5 *μ*M), and sunitinib (0.01, 0.1, and 1 *μ*M) for 30 min, and stimulated with the serum (the same final concentration as mentioned previously) for 5 min. Finally, cells were harvested and phosphorylation of AKT, ERK1/2, and mTOR was determined by Western blotting. Because serum-stimulated cells had a conspicuous positive expression of p-AKT, p-ERK1/2, and p-mTOR, cells treated with the serum could naturally be taken as a positive control. Upon the determination of the optimal stimulation time and concentration of the antagonists, the cells were pretreated with LY294002 (10 *μ*M), U0126 (10 *μ*M), AZD8055 (1 *μ*M), sunitinib (1 *μ*M), or vehicle for 30 min and incubated with the serum for another 5 min. Cells were then collected and washed twice with ice-cold PBS. The lysates were prepared with cell lysis buffer supplemented with complete protease inhibitor cocktail. Afterwards, the lysates were centrifuged at 10000 ×g at 4°C for 10 min and the supernatants were quantitatively detected for total protein by using the BCA assay. Frozen tissues were homogenized on ice in a tissue protein extraction reagent according to the manufacturer's protocol. The soluble protein concentrations were determined before loading.

### 2.5. Western Blot Analysis

Western blotting was performed as described before with minor modifications [22]. Proteins were separated on 8%–12% SDS-PAGE, transferred to polyvinylidene difluoride membrane, and blocked with milk. Membranes were incubated with primary antibodies for p-AKT, p-ERK1/2, p-mTOR, and *β*-actin overnight at 4°C and then with horseradish peroxidase-conjugated secondary antibodies at room temperature for 1 hour. The bands were visualized on an enhanced chemiluminescence (ECL) detection system (GE Healthcare, Little Chalfont, UK).

### 2.6. Cell Proliferation Assay

Cells were plated into 96-well plates at a density of 4 × 10^4^ cells/well in a final volume of 200 *μ*L and cultured for 4 days. Afterwards, cells were incubated in fresh 15% FBS DMEM with or without LY294002 (10 *μ*M), U0126 (10 *μ*M), AZD8055 (1 *μ*M), and sunitinib (1 *μ*M) for 48 hours. Culture medium was removed and MTT solution was added to the plates. Cells were incubated at 37°C for 1 hour. Then, the medium was removed and DMSO was added to dissolve the purple formazan crystals for three hours. Finally, the plates were read on a microplate reader (Bio-TEK Instruments, Vermont, USA) at 490 nm with a reference filter at 630 nm. For each PPGL sample (*n* = 10), 5 sets (or groups) of wells were set up, each set having 4 wells.

### 2.7. Statistical Analysis

Data were statistically analyzed by employing the SPSS 14.0 software package. The data of Western blotting were expressed as means ± standard error, and the significance of differences was evaluated by paired samples *t*-test. Meanwhile, the data from cell proliferation assay were presented as means ± standard deviation, and significance of differences was assessed by using independent samples* t*-test. *P* < 0.05 was considered to be statistically significant.

## 3. Results

### 3.1. Phosphorylation of AKT, ERK1/2, and mTOR in PPGL Tissues

**Figure 1:**
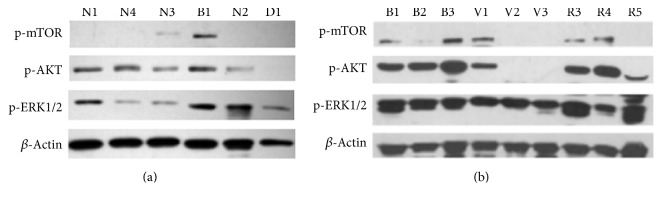
Phosphorylation of AKT, ERK1/2, and mTOR in PPGL tissues. (a) Phosphorylation of AKT, ERK1/2, and mTOR in 6 PPGLs used in* in vitro* experiments. (b) Phosphorylation of AKT, ERK1/2, and mTOR in 9 PPGLs from patients with different gene mutations. *β*-Actin was used as a loading control. N: PPGL without gene mutation of SDHB, SDHC, SDHD, VHL, and RET; B: PPGL with SDHB mutation; D: PPGL with SDHD mutation; V: PPGL with VHL mutation; R: PPGL with RET mutation.

Whether PI3K/AKT/mTOR and MAPK/ERK pathways are activated in PPGLs remains unknown. Therefore, we examined the phosphorylation of AKT, ERK1/2, and mTOR in PPGL tumor samples. First, phosphorylation was detected in 6 samples used for* in vitro* experiments, and the result revealed that p-AKT, p-ERK1/2, and p-mTOR varied greatly with different human PPGL tissues (Figure 1(a)). To clarify whether such variation was dictated by genetic background of the tumors, 9 PPGLs with different gene mutations were analyzed.  Figure 1(b) showed that ERK1/2 was activated in all PPGLs and AKT and mTOR were activated in most of the tumors. Phosphorylation of AKT and ERK1/2 appeared to be more pronounced in SDHB-related PPGLs than in VHL-related PPGLs. mTOR phosphorylation was detected in all the three SDHB-related PPGLs, while it was detected only in one VHL-related tumor. RET-related PPGLs showed conspicuous activation of AKT and ERK1/2, and mTOR activation was detected in two of the three RET-related tumors.

### 3.2. Effect of Pathway Inhibitors on Phosphorylation of AKT, ERK1/2, and mTOR

**Figure 2:**
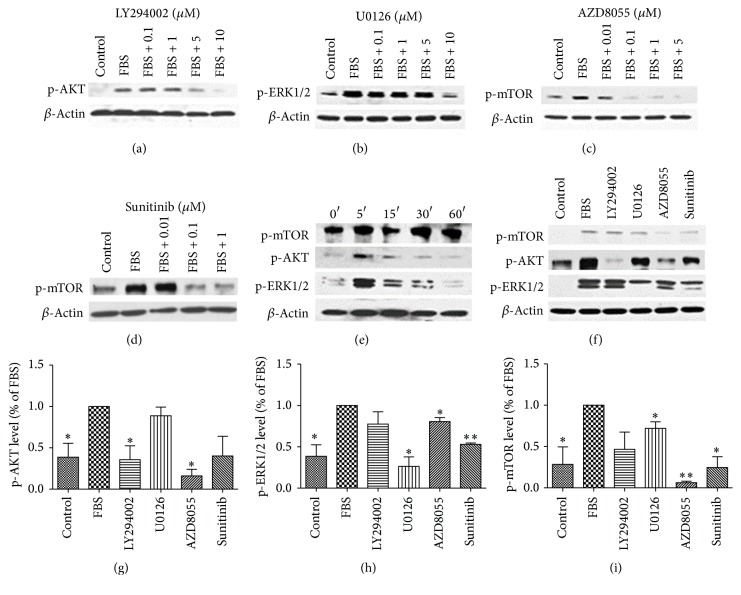
Effects of pathway inhibitors on phosphorylation of AKT, ERK1/2, and mTOR in human PPGL cells. (a–d) Dose-dependent inhibition of p-AKT (a), p-ERK1/2 (b), and p-mTOR (c, d) by pathway inhibitors (LY294002 (a), U0126 (b), AZD8055 (c), and sunitinib (d)). (e) Time course of phosphorylation of AKT, ERK1/2, and mTOR induced by the serum. (f) Effects of LY294002 (10 *μ*M), U0126 (10 *μ*M), AZD8055 (1 *μ*M), and sunitinib (1 *μ*M) on the phosphorylation of AKT, ERK1/2, and mTOR in human PPGL cells. *β*-Actin was used as a loading control. Because serum-stimulated cells had a conspicuous positive expression of p-AKT, p-ERK1/2, and p-mTOR, cells treated with the serum could naturally be taken as a positive control. The experiment was repeated three times. (g, h, i) The histograms represent the densitometric results of the phosphorylation from three independent experiments. ERK1/2, AKT, and mTOR phosphorylation in FBS group was taken as 100%. ^*∗*^ *P* < 0.05 versus FBS group; ^*∗∗*^ *P* < 0.01 versus FBS group.

Figures 2(a)–2(d) show that LY294002, U0126, AZD8055, and sunitinib dose-dependently inhibited AKT, ERK1/2, or mTOR phosphorylation at the optimal concentration of 10 *μ*M (for LY294002 and U0126) and 0.1–1 *μ*M (for AZD8055 and sunitinib). Stimulation of the cells with serum for different periods of time caused time-dependent phosphorylation of ERK1/2 and AKT with the maximum effect occurring at 5 min. Additionally, mTOR was highly activated 5 min after the stimulation (Figure 2(e)). Treatment with LY294002 (10 *μ*M) reduced only AKT phosphorylation. U0126 (10 *μ*M) inhibited ERK1/2 phosphorylation and downregulated the activation of mTOR. AZD8055 (1 *μ*M) significantly inhibited the activation of AKT, ERK1/2, and mTOR. Treatment with sunitinib (1 *μ*M) in PPGL cells dramatically downregulated both ERK1/2 and mTOR phosphorylation. The findings suggested that there was a cross talk between MAPK/ERK and PI3K/AKT/mTOR signaling pathways (Figures 2(f)–2(i)).

### 3.3. Effect of Pathway Inhibitors on the Proliferation of Human PPGL Cells

**Figure 3:**
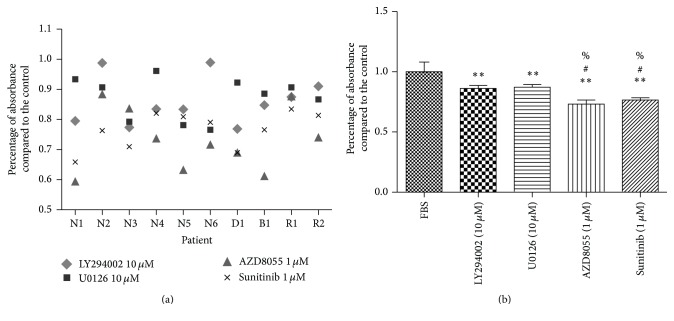
Effect of pathway inhibitors on cell proliferation in human PPGL cells. Experiments were performed in four independent wells for each group (*n* = 4) and repeated in ten PPGLs (*N* = 10). The results were represented in the scatter diagram (a) and column chart (b). ^*∗∗*^ *P* < 0.01* versus* control group; ^#^ *P* < 0.05* versus* LY294002 group; ^%^ *P* < 0.05* versus* U0126 group. N: PPGL without gene mutation of SDHB, SDHC, SDHD, VHL, and RET; B: PPGL with SDHB mutation; D: PPGL with SDHD mutation; R: PPGL with RET mutation.

To determine the roles of the PI3K/AKT/mTOR and MAPK/ERK signaling pathways in the survival of human PPGL cells, human PPGL cells were, respectively, treated with corresponding inhibitors of the pathways. As shown in Figure 3, LY294002 (10 *μ*M), U0126 (10 *μ*M), AZD8055 (1 *μ*M), and sunitinib (1 *μ*M) inhibited PPGL cell proliferation in ten primary cultures of tissues from different patients, including four patients with gene mutations (2 with RET mutation, 1 with SDHD mutation, and 1 with SDHB mutation) (Figures 3(a) and 3(b)). Only in two unrelated cultures did the cells fail to respond to the treatment of LY294002. Cells in another three unrelated cultures did not respond to U0126 (Figure 3(a)). mTORC1/2 inhibitors and sunitinib exerted stronger inhibiting effect on cell growth compared to PI3K inhibitor and MEK1/2 inhibitor (Figure 3(b)).

## 4. Discussion

Understanding the changes in signaling pathways involved in PPGLs can help us find new targets for tumor treatment. In this study, we looked into the role of the signaling pathways in the pathogenesis by blocking related pathways with their respective inhibitors, with an attempt to understand the impact of these pathways on the survival of tumor cells.

PC12 cells, originating from rat pheochromocytoma, have been widely employed as a model for the study of pathogenesis of PPGLs. On the other hand, some researchers also used a newly established mouse pheochromocytoma cell line from heterozygous NF1 gene knockout mice [23]. Although they are all of pheochromocytoma origin, they might not necessarily undergo the molecular and functional changes that true human catecholamine-producing tumors go through* in vivo*. In our study, we used the primary culture of human PPGL cells and presumably the conditions could better mimic the environment of human PPGLs.

This study showed that separately blocking PI3K/AKT/mTOR and MAPK/ERK signaling pathways was able to inhibit the proliferation of human PPGL cells from patients with different gene backgrounds. Furthermore, Western blot studies showed that PI3K/AKT/mTOR and MAPK/ERK signaling pathways were activated when PPGL cells were treated with serum, suggesting that these signaling pathways are functionally correlated in tumorigenesis. To our knowledge, this is the first experimental evidence showing a strong correlation between the tumorigenicity of pheochromocytoma cells and the activity of these signaling molecules in a human primary cell culture model.

Extensive cross talk between PI3K/AKT/mTOR and MAPK/ERK pathways has been previously documented. These two signaling pathways have been proved to be implicated in the cross talk between insulin and Ang II systems. ERK phosphorylation stimulated by Ang II inhibited insulin-induced activation of the IRS-1/PI3K/AKT pathway [24]. Our study demonstrated that inhibition of MEK resulted in decreased phosphorylation of mTOR. Also, specific inhibition of mTOR activation by AZD8055 reduced phosphorylation of both AKT and ERK. These results supported the notion that PI3K/AKT/mTOR and MAPK/ERK signaling pathways are not independent but interactive. Compensatory activation of PI3K/AKT and MAPK signaling pathways has been demonstrated previously [25]. In human neuroendocrine tumor cell lines, blockage of Raf inhibited ERK1/2 phosphorylation but strongly induced AKT phosphorylation, suggesting that there exists a compensatory feedback loop between these two pathways [26]. Conversely, the upregulation of PI3K signaling pathway induced by epidermal growth factor caused MEK inhibition [27]. However, this compensatory feedback loop was not observed in our study. Moreover, it is well documented that inhibition of both MEK/ERK and mTOR substantially enhanced their antitumor effects on prostate cancer both* in vitro* and* in vivo *[28]. A recent study demonstrated that treatment with NVP-BEZ23 (PI3K/mTORC1/2 inhibitor) in combination with lovastatin (ERK1/2 inhibitor) exerted a significant additive antitumor viability in mouse PPGL cell lines [29]. Given these findings, a question will present itself as to whether concurrent MAPK and mTOR inhibition may result in substantially enhanced antitumor effects on human PPLG cells.

mTOR serves as a connector between PI3K/AKT signaling and critical downstream pathways and is a master regulator of cell proliferation and survival [30]. Activated AKT promotes mTORC1 signaling pathway by decreasing TSC1/2 inhibition [19], while mTOR-C1 inhibition alone leads to compensatory activation of AKT signaling pathway mediated by mTOR-C2 [31]. In the present study, mTORC1/2-mediated inhibition of human PPGL cell proliferation was the strongest as compared to PI3K- and MAPK-mediated inhibition, indicating that mTOR might be a major regulator of cell proliferation. We also found that inhibition of both mTOR-C1 and mTOR-C2 strongly downregulated AKT activation, and the finding was consistent with the result observed in rat pheochromocytoma PC12 cell tumor model, which showed that PP242, dual mTOR complex 1 and 2 inhibitor, but not rapamycin, dramatically inhibited tumor growth, suggesting that mTORC-2 inhibition plays an important role and could disturb the mTORC1-dependent negative feedback loops [32]. Therefore, inhibition of both mTOR-C1 and mTOR-C2 might be a novel therapeutic approach for PPGLs and might overcome the problems associated with the use of mTOR-C1 inhibitor alone. A recent study, by separately transfecting with mTOR-C1, mTOR-C2, and mTOR1/2 small interfering RNA, found that targeted inhibition of mTORC-2 or mTORC1/2, but not mTOR-C1, could effectively prevent proliferation, migration, and invasion and promote apoptosis of PC12 cell line [33]. These data suggest that targeting mTOR-C2 might be a novel alternative for the treatment of PPGLs. Nonetheless, mTORC2-specific inhibitors are not available and more studies are warranted to confirm the speculation.

Sunitinib is an small-molecule multitargeting inhibitor of receptor tyrosine kinase (RTK), with antiangiogenic and antitumor activity that primarily targets vascular endothelial growth factor receptors (VEGFRs) [34, 35]. It has been found that PI3K/AKT, protein kinase C (PKC) family, and MAPK/Ras signaling cascades played important roles in RTK-activation-related cancer development [36]. Our results revealed that sunitinib was able to block the proliferation of human PPGL cells by inhibiting p-mTOR in the PI3K/AKT/mTOR signaling pathway and p-ERK in the MAPK signal pathway. These findings suggest that inhibition of multiple cell signaling pathways contributes to the antitumor effect of sunitinib. Saito et al. demonstrated that sunitinib directly inhibited mTOR-C1 signaling pathway, which in turn led to apoptosis of PC12 cells [37]. Denorme et al. exhibited a dual inhibitory effect of sunitinib on both angiogenesis and tumor cell viability in a pheochromocytoma xenograft model [38]. Moreover, it has been shown that the suppression of mTOR-C1 signaling pathway enhanced sunitinib-induced autophagy in rat pheochromocytoma PC12 cells [39]. Clinical case reports demonstrated that sunitinib appeared to be effective for the treatment of malignant PPGLs [40]. A study reported that about half of seventeen patients with progressive metastatic PPGLs treated with sunitinib showed favorable clinical results [41]. These findings, together with our present results, suggest that sunitinib promises to be an effective agent that directly, though partially, inhibits PI3K/AKT/mTOR and MAPK pathways.

In this study, we also examined the activation of PI3K/AKT/mTOR and MAPK signaling pathways in PPGLs. We found that AKT, ERK, and mTOR were activated in most PPGLs. Furthermore, their activation appeared to be more pronounced in SDHB-related PPGLs than in VHL-related PPGLs but larger sample studies are needed to further confirm the result. It has been reported that SDHB-mutated tumors possessed high metastatic potential [42]. Therefore, the difference in PI3K/AKT and MAPK/ERK signaling pathways observed in this study might be associated with the malignant nature of SDHB-associated PPGLs. In this study, we observed a wide variation in activation states in VHL- and RET-associated PPGLs, which has yet to be explained in further studies.

As far as we know, this is the first study exploring the molecular pathways in primary human PPGL cells. However, the study has some limitations. Firstly, up to 19 possible susceptibility genes are associated with the pathogenesis of PPGLs and not all susceptibility genes were detected in our series. Secondly, the sample size of our study was relatively small. Because the sample size of the tumors with gene mutations is not large enough to make a comparison among genotypes, we could not tell exactly whether any of the 4 inhibitors work better in any of the genotypes. Thirdly, we were not able to perform apoptosis and invasion/migration experiments in this study as we did not have enough cells to conduct these assays. Finally, we currently do not have any data concerning the mRNA transcription levels for these pathways.

In conclusion, PI3K/AKT/mTOR and MAPK/ERK signaling pathways play vital roles in human PPGL cell growth. AKT, ERK, and mTOR are activated in most PPGLs. In view of the cross talk between PI3K/AKT/mTOR and MAPK/ERK signaling pathways, we are led to believe that inhibition of multiple pathways may be a novel therapeutic approach for the treatment of PPGLs.

## Figures and Tables

**Table 1:** Patients' information.

Patient	Gender	Age at diagnosis (years)	Location	Genetic background	Tumor size (cm)	Urinary norepinephrine (*μ*g/24 h)	Urinary epinephrine (*μ*g/24 h)	Urinary dopamine (*μ*g/24 h)
N1	F	72	Adrenal	N	3.0	22.4	3.7	156.6
N2	F	57	Adrenal	N	4.0	19.3	1.9	181.3
N3	F	40	Adrenal	N	4.7	16.9	1.5	67.9
N4	M	59	Adrenal	N	6.3	100.9	2.8	140.1
N5	F	26	Adrenal	N	4.0	20.4	2.3	135.7
N6	M	40	Adrenal	N	5.0	177.1	1.1	256.6
D1	F	47	Adrenal	SDHD	8.0	215.0	2.8	311.1
B1	M	26	Retroperitoneal	SDHB	5.0	138.9	2.3	1046.5
B2	M	62	Adrenal	SDHB	3.7	15.4	1.9	177.4
B3	F	48	Retroperitoneal	SDHB	9.0	121.4	1.8	147.1
V1	M	21	Adrenal	VHL	7.5	937.5	2.3	127.3
V2	F	34	Adrenal and retroperitoneal	VHL	4.5 (adrenal)/4.0 (retroperitoneal)	239.2	13.3	153.8
V3	M	8	Adrenal	VHL	4.0	214.1	4.6	98.3
R1	F	33	Adrenal	RET	3.7	13.9	2.7	237.6
R2	F	30	Adrenal	RET	5.0	21.2	5.3	456.2
R3	M	49	Adrenal	RET	2.4	20.8	3.5	326.3
R4	F	27	Adrenal	RET	7.3	20.5	3.0	163.2
R5	F	45	Adrenal	RET	6.9	315.3	2.6	268.8

N: PPGL without gene mutation of SDHB, SDHC, SDHD, VHL, and RET; M: male; F: female. Reference range: urinary norepinephrine 16.7–40.7 *μ*g/24 h; urinary epinephrine 1.7–6.4 *μ*g/24 h; urinary dopamine 120.9–330.6 *μ*g/24 h.
